# Effect of Collagen Tripeptide and Adjusting for Climate Change on Skin Hydration in Middle-Aged Women: A Randomized, Double-Blind, Placebo-Controlled Trial

**DOI:** 10.3389/fmed.2020.608903

**Published:** 2021-01-11

**Authors:** Young Jin Tak, Dae Keun Shin, Ae Hyang Kim, Jun Il Kim, Ye Li Lee, Hyun-Chang Ko, Yong-Woo Kim, Sang Yeoup Lee

**Affiliations:** ^1^Department of Family Medicine and Biomedical Research Institute, Pusan National University Hospital, Busan, South Korea; ^2^Amicogen Inc. Biotech R & D Center, Jinju, South Korea; ^3^Integrated Research Institute for Natural Ingredients and Functional Foods, Yangsan, South Korea; ^4^Department of Dermatology, Pusan National University Yangsan Hospital, Yangsan, South Korea; ^5^Department of Radiology, Pusan National University Yangsan Hospital, Yangsan, South Korea; ^6^Family Medicine Clinic and Research Institute of Convergence of Biomedical Science and Technology, Pusan National University Yangsan Hospital, Yangsan, South Korea; ^7^Department of Medical Education, Pusan National University School of Medicine, Yangsan, South Korea

**Keywords:** collagen tripeptide, skin hydration, skin elasticity, skin wrinkling, middle aged women, climate change

## Abstract

**Introduction:** Although collagen is widely used in various forms as a functional ingredient in skin care products, the effect of oral supplementation of collagen tripeptides (CTPs) on human skin is unclear. Moreover, the majority of the positive outcomes of CTP reported so far have not considered the effect of weather conditions. Therefore, we tested the effect of CTP and adjusting for climate change on skin properties in middle-aged women.

**Materials and Methods:** A randomized controlled trial was conducted with 84 women between 40 and 60 years of age. Participants were randomized to receive placebo or 1,000 mg CTP daily for 12 weeks. CTP was prepared from the skin of *Nile Tilapia* by the digestion method using collagenase from non-pathogenic bacteria of the genus *Bacillus*. Skin hydration, wrinkling, and elasticity were assessed at baseline and after 6 and 12 weeks with adjustments for temperature, humidity, and ultraviolet A exposure during the evaluation time using weather data from the regional meteorological office.

**Results:** Of the 82 participants, 74 completed the trial without adverse effects. Compared with the control group, trans-epidermal water loss was reduced more in the CTP group after 12 weeks (*P* < 0.05). At 12 weeks, even after adjustment for humidity, temperature, and UVA in the region, the difference of the two groups in TEWL remained statistically significant (adjusted for humidity and temperature, *P* = 0.024; adjusted for UVA, *P* = 0.032; adjusted for temperature, high temperature, and ultraviolet A, *P* = 0.031). In terms of skin hydration, more improvement was evident in the CTP group than in the control group. In the subgroup analysis, subjects under 50 years of age showed a significant improvement in total score and moisture in the subjective skin improvement questionnaire after taking CTP for 12 weeks. Application of CTP was well-tolerated, and no notable adverse effect was reported from both groups.

**Discussion:** Our findings suggest that oral ingestion of CTP from the Skin of Nile Tilapia (*Oreochromis niloticus*) is well-tolerated and helps reduce water loss in in middle-aged women.

**Clinical Trial Registration:** www.clinicaltrials.gov/, Identifier: NCT03505684.

## Introduction

In the natural process of aging, elastin fibers and collagen in the skin decrease, which eventually results in skin aging, fine lines, and deep wrinkles (1). The amount of glycosaminoglycans (GAGs) in the epidermis and dermis decline with age, leading to a decrease in the capacity to retain moisture within the skin and an increase in skin dryness (2). Collagen is the major structural constituent of the dermal extracellular matrix (ECM), which accounts for more than 70% of the dry weight of the normal human skin dermis (3).

New insights into the effects of the oral intake of biologically active compounds on skin properties have led to the development of nutritional supplements to benefit human skin (4). For instance, some dietary supplements with antioxidant properties reportedly influence the skin *via* secondary messengers. Alternatively, in the process of digestion, they can traverse the gastrointestinal tract, cross the intestinal barrier, and reach the skin, potentially in an active form, *via* the blood stream. Blood containing these bioactive compounds continuously replenishes the skin and all skin compartments, including the epidermis, dermis, subcutaneous fat, and sebum (5). Among these compounds, collagen has been widely used as a material in food, cosmetic, and pharmaceutical industries due to its biological and functional properties (6). In particular, collagen peptides derived from sea fish have been recognized as a dietary supplement that is useful for treating high blood pressure (7), muscle damage (8), lipid and glucose control (7, 9), and weight management in overweight individuals (10).

Collagen has a triple helix configuration (Gly-X-Y)n in which X and Y are mostly proline (Pro) and hydroxyproline (Hyp) (11). After consumed, Gly-Pro-Hyp is partially hydrolyzed by the brush-border membrane of the intestinal epithelium-bound aminopeptidase N to eliminate Gly (12). Thereafter, Pro-Hyp, a major active constituent of collagen-derived peptides, can be transported into small intestinal epithelial cells *via* the H+-coupled oligopeptide transporter (PEPT-1) (13). A higher level of Gly-Pro-Hyp in plasma was detected after the oral intake of low molecular weight collagen hydrolysates or collagen tripeptide (CTP) compared to high molecular weight collagen peptide (14). In addition, previous studies showed that Gly-Pro-Hyp and Pro-Hyp were stable in gastrointestinal fluid and plasma without being decomposed by gastric acid and enzymes, pancreatin, or plasma peptidases (14). These results indicate that oral intake of CTP can be an efficient approach to taking bioactive peptides owing to the enzymatic stability and intestinal permeability of Gly-Pro-Hyp and Pro-Hyp.

Evidence regarding the benefits of CTP continues to accumulate. In a recent randomized controlled trial, the oral intake of a low-molecular-weight collagen peptide (LMWCP) improved hydration, elasticity, and wrinkling in the skin of middle-aged women (15). However, the effect of LMWCP, evaluated by measuring various skin properties, was assessed without considering the influence of the temperature, humidity, and ultraviolet A (UVA) at the time of testing. Given that climatic factors can affect the overall skin texture, it is prudent to evaluate the effect of CTP on skin health by analyzing skin parameters after adjusting for the temperature, humidity, and UVA of the region and the season that the subjects were tested in. This randomized, double-blind clinical trial assessed whether the oral administration of CTP derived from fish skin of Nile Tilapia could still improve skin condition among middle-aged women after adjustments for the weather conditions.

## Materials and Methods

### Study Design and Ethical Aspects

The study was designed as a randomized, placebo-controlled, double-blind clinical trial. All subjects gave their informed consent for inclusion before they participated in the study. The study was conducted in accordance with the Declaration of Helsinki, and the protocol was approved by the Ethics Committee of the Institutional Review Board at Pusan National University Yangsan Hospital (IRB No. 02-2017-033, November 6, 2017). This trial is registered with ClinicalTrials.gov (NCT03505684, April 23, 2018).

### Study Participants

Apparently healthy candidates were recruited through an advertisement at a tertiary hospital in Yangsan-si, South Korea. Eligible subjects included those between the ages of 40 and 60 years with a transepidermal water loss (TEWL) score ≥4 measured using a Tewameter. Additionally, the candidates were required to apply the same skin care products (topical and oral supplements) as those used 4 weeks before the enrollment and did not receive any other skin care procedures during the 12-week study period. The main exclusion criteria were as follows: use within the 3 months preceding enrollment of any medication or supplements that could change in skin properties, including retinoids, steroids, and any other hormonal products, history of skin hypersensitivity and allergic reaction to any ingredients and sunlight, any skin improvement procedure within the prior 6 months, alcohol abuse, smoking cessation within 3 months of enrollment, uncontrolled blood pressure, blood glucose, or gastrointestinal symptoms, aspartate aminotransferase (AST) or alanine aminotransferase (ALT) serum level >80 mg/dL, or creatinine (Cr) level >1.5 mg/dL, pregnancy or breastfeeding, allergy to any study ingredient, and intention to move away during the period of the study. In addition, for safety reasons, candidates diagnosed with cardiovascular diseases or any cancer during the 6 months prior to study commencement were also excluded. The subjects were considered to drop out or discontinue participation in the trial and were excluded from the per protocol analysis according to the following criteria: not taking the test product or placebo for 5 consecutive days, failure to attend follow-up assessments, poor compliance (<80%), excessive alcohol consumption or sunlight exposure, and relocation elsewhere during the period of the study. Compliance was assessed by counting the remaining capsules at every visit. Eleven participants met the exclusion criteria and three participants declined to participate.

### Randomization

**Figure 1:**
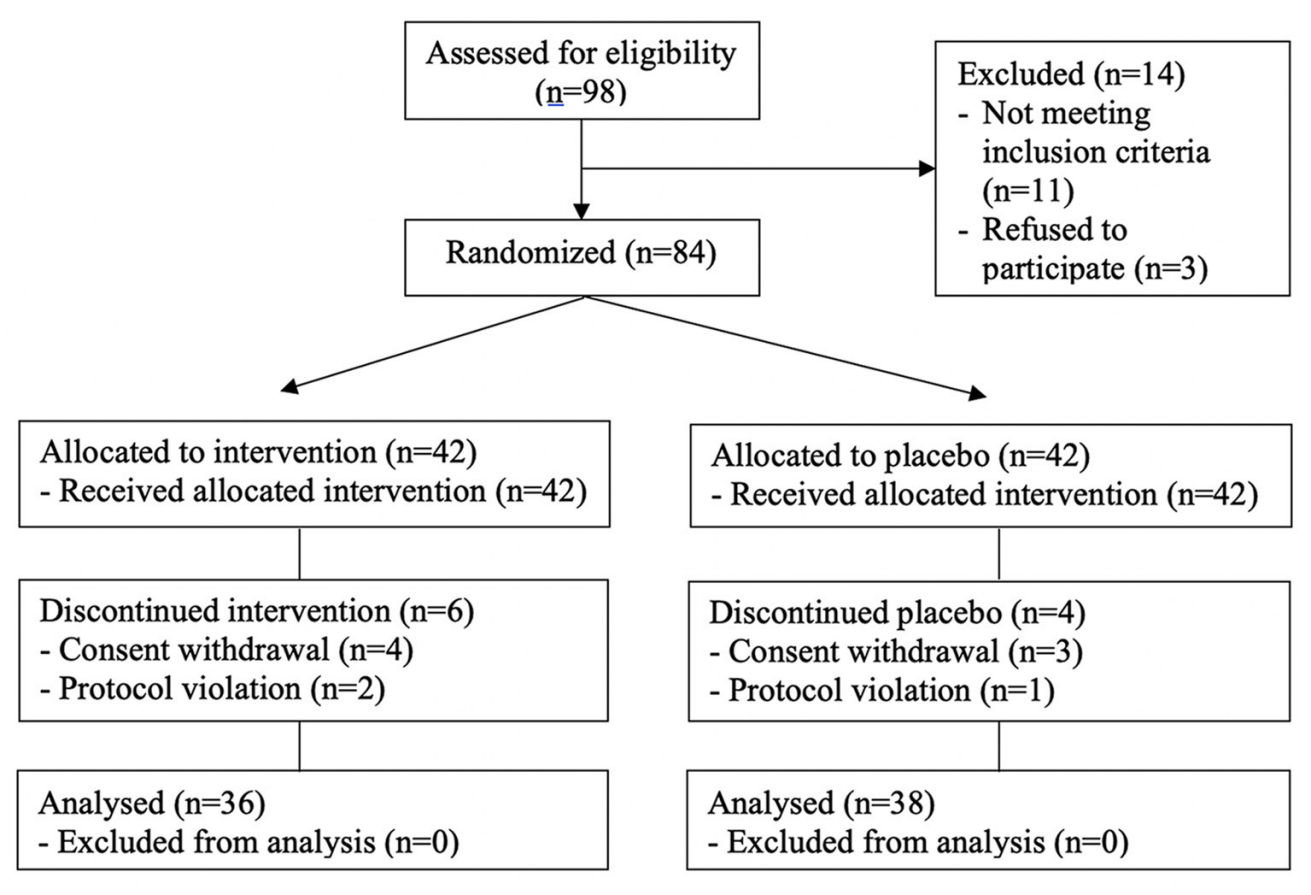
CONSORT flow diagram.

Ninety-eight participants were recruited for screening and 84 (85.7%) participants were finally enrolled after undergoing baseline measurements. They were randomly assigned to one of the intervention group with CTP (IG) and the control group with placebo (CG) through block randomization using randomized numbers and given identification numbers on recruitment. The IG (*n* = 42) received 1,000 mg of CTP supplement (250 mg each capsule) per day. The CG (*n* = 42) received a placebo (Figure 1). Randomization codes were created by an expert in statistics using nQuery Advisor 7.0. Those who were responsible for deciding on study eligibility and conducting the measurements were kept unaware of the results of the randomization throughout the whole study process. Every participant was asked to visit the center four times in total (visit 1; for screening, visit 2; randomization and start taking supplements, visit 3; 6 weeks after intervention, visit 4; 12 weeks later).

### Test Product and Placebo

CTP was prepared from the skin of Nile Tilapia (*Oreochromis niloticus*) by collagenase digestion (Collagen-Tripep20S; Amicogen Inc., Jinju, Gyeongsangnam-do, South Korea) from non-pathogenic bacteria of the genus *Bacillus*. The average molecular weight of the total CTPs was 500 Da with a 20% CTP content including 3.2% Gly–Pro–Hyp. Gly–Pro–Hyp was proven to be stable and bioavailable *in vivo* (14, 15). Additionally, its cutaneous hydration effect was demonstrated histologically by the enhanced gene and protein expressions in mice (16). The dosage of CTP applied to the subjects had been determined based on the results from previous animal studies where mice fed CTP daily (333 mg/kg) had shown a significant improvement in skin hydration and wrinkling without any adverse events (17, 18). Thus, 1,000 mg of collagen hydrolysate was considered appropriate for humans with an average body weight of 60 kg according to the guidance for estimating the maximum safe starting dose in initial clinical trials for therapeutics in adult healthy volunteers (19). In addition, in studies similar to our study, subjects were given a dose of between 1.0 and 3.0 g/day (15, 20–22). Two capsules (250 mg per capsule) of CTP were taken twice a day in the morning and evening by the IG subjects (total of four capsules each day) for 12 weeks. CG subjects were given the placebos with the same protocol and duration. Placebo consisted of 170 mg (68.2%) of maltodextrin and 80 mg (31.8%) of dextrin. In our preliminary study, plasma titer level of tripeptide and kinetics of the product were demonstrated (14).

### Measurements of Efficacy

Prior to assessment for skin properties at every visit, the participants stayed for 30 min in the same room with controlled temperature (23 ± 1°C) and humidity (45 ± 5%). The primary efficacy variable was the change from the baseline in TEWL at 6 and 12 weeks of CTP or placebo use. The secondary outcome parameters involving skin hydration, elasticity, wrinkling and the participants' self-assessments were assessed at every visit.

#### Skin Transepidermal Water Loss

Three measurements were performed on the skin of the 10 cm frontal area from the antecubital fossa using an evaporimeter (Tewameter TM300; Courage + Khazaha Electronic GmbH, Köln, Germany) (23). The average value was reported in the analysis.

#### Skin Hydration

Hydration of the external layer of the epidermis (stratum corneum) was measured on the forehead capacitively with a corneometer (Corneometer CM 825; Courage + Khazaha Electronic GmbH, Köln, Germany) (24). For each measurement time, at least five measurements at different locations in the test area on the forehead were performed, then the average of three values excluding maximum and minimum readings were used for the analysis.

#### Skin Elasticity

For the assessment of skin elasticity, a cutometer (Cutometer MPA 580; Courage + Khazaha Electronic GmbH, Köln, Germany) was used (24). For each measurement, skin elasticity at three different places of the test area (forearm) was assessed and the values were averaged.

#### Skin Wrinkling

Measurement of skin wrinkling was evaluated on both sides of the crow's-feet area using a visiometer (Skin-Visiometer SV 600; Courage + Khazaka electronic GmbH, Köln, Germany) (25). The visual grade was evaluated on a scale of global photodamage scoring system (25) by a blinded dermatologist.

#### Questionnaire

After 6 and 12 weeks, the study subjects completed a questionnaire and subjectively assessed their perception of improvement in skin properties with time. Their responses were based on a 5-point Likert scale (1, much worse; 2, worse; 3, unchanged; 4, improved; 5, much improved). In addition, any unpleasant experience related to the study supplement was also reported.

### Safety Evaluation

Blood pressure and heart rate were tested three times in the sitting position after a 10-min rest using a model BP-203 RV II device (Colin Corp., Aichi, Japan). The average measurement was recorded. Body weight and height were measured using a digital scale and stadiometer (BSM370; Biospace Co. Ltd., Seoul, South Korea), with patients wearing a light gown without shoes. Following a 4 h fast, blood samples were collected at baseline and at 6 and 12 weeks after start of the study. Serum AST, ALT, total bilirubin, glucose, and Cr were measured using a model TBA200FR biochemical analyzer (Toshiba Co. Ltd., Tokyo, Japan). Reports of any other adverse events or unpredicted allergic reactions were collected throughout the study.

### Climate Data

The data on the temperature, humidity, and UVA of Kyungsang province and Busan, which were the regions where all participants resided during the entire study period (from January through October) were obtained from the local meteorological office.

### Statistical Analyses

nQuery Advisor software version 7.0 (Cork, Ireland) was used to calculate sample size. The sample size of the study was calculated based on Choi et al. (21), who tested the effects of CTP supplement on skin properties. The estimated sample size was 34 subjects per group for 80% power to detect a difference of 3.53 in the mean TEWL levels, assuming a standard deviation of 5.0 in the primary outcome variable and an alpha error of 5%. Then, the sample size was adjusted to 42 participants per group to allow for 20% dropouts. Intent to treat (ITT) was the primary analysis for comparisons of outcomes between the two groups with multiple imputation of missing data (*n* = 84). A per-protocol (PP) analysis was also performed (*n* = 71) to assess effectiveness of the supplementation. The Shapiro–Wilk's test was performed to test the normality assumption. Intergroup comparisons of baseline characteristics and their changes at weeks 6 and 12 of the trial were performed using the two-sample *t*-test for continuous variables (or Mann-Whitney's U test in case of valuables showing non-normal distributions) or the Chi-square test for categorical variables. Intragroup comparisons were conducted using the paired *t*-test for continuous variables (or Wilcoxon signed rank test in case of valuables showing non-normal distributions). In addition, the subgroup analysis was performed based on the age. A repeated measures analysis of covariance was performed to compare intergroup differences in outcomes after adjustment for temperature, humidity and UVA of the day when parameters were checked. A *P* < 0.05 was considered statistically significant. SPSS Statistics for Windows Version 22.0 (IBM Corp., Armonk, NY) was used for the analysis.

## Results

### CONSORT (Consolidated Standards of Reporting Trials) Flow Diagram and Baseline Characteristics of the Subjects

The flow of subjects through the controlled interventional trial is depicted in a CONSORT conform diagram (26) (Figure 1). Eighty-four subjects aged 40–60 years were statistically analyzed. The mean age in the IG (*n* = 42) was 48.0 ± 5.9 years and in the CG (*n* = 42), 49.9 ± 6.5 years. Four participants in the IG and three in the CG withdrew consent for personal reasons that were not considered associated with the trial. Additionally, three subjects (on two in the IG and one in the CG) were excluded due to the protocol violation. The characteristics of these 10 participants were similar to those of the others who completed the study. Compliance exceeded 88% in both the IG and CG. Hence, ITT population and PP population were 84 and 71, respectively. No statistically significant intergroup differences were observed for age, body weight, alcohol drinking, and smoking at baseline (Table 1). The double-blind requirement was well-maintained throughout the study.

**Table 1:** Baseline characteristics of the study subjects.

	**Intention to treat population**			**Per protocol population**		
	**CG (*n* = 41)**	**IG (*n* = 41)**	***P***	**CG (*n* = 38)**	**IG (*n* = 36)**	***P***
Age (years)	49.9 ± 6.5	48.0 ± 5.9	0.176	49.5 ± 6.4	47.9 ± 6.0	0.303
Weight (kg)	56.7 ± 6.8	57.4 ± 8.9	0.813	56.4 ± 7.0	57.1 ± 9.2	0.864
Height (cm)	157.9 ± 5.7	157.6 ± 4.9	0.807	158.1 ± 5.7	157.5 ± 4.9	0.614
Alcohol drinking (%)	2 (4.8)	4 (9.8)	0.396	2 (5.3)	3 (8.3)	0.599
Smoker (%)	0 (0)	0 (0)	–	0 (0)	0 (0)	–

*CG, control group with placebo; IG, intervention group with collagen tripeptide. Alcohol drinking was defined as consumption of alcohol with an average of five cups or more, more than two times a week. Data are presented as mean ± standard deviation or number (%). Shapiro–Wilk's test was employed for test of normality assumption. Mann–Whitney's U test for age, weight, two-sample t-test for height, Chi-square test for alcohol drinking*.

### Changes in Skin Properties

#### Skin Parameters

**Figure 2:**
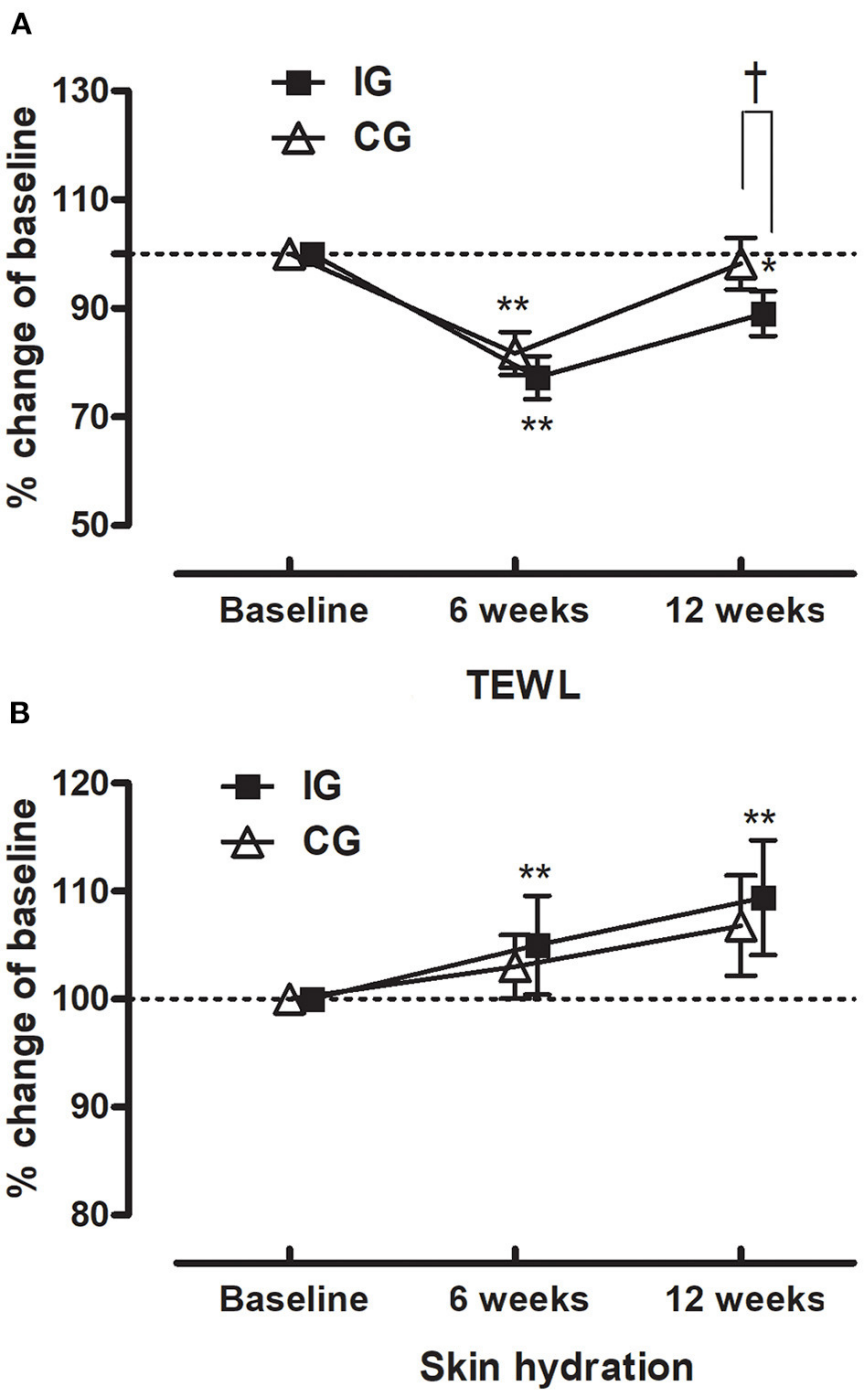
Changes in skin parameters in individuals receiving collagen tripeptide or placebo. **(A)** Changes in transepidermal water loss (TEWL). **(B)** Changes in skin hydration. *P < 0.05, **P < 0.01 by paired *t*-test comparisons with baseline values. *P* < 0.05 by two-sample *t*-test comparisons between values in the intervention group and the control group.

After 6 and 12 weeks of the trial, both groups showed decreased water loss with a greater reduction in TEWL in the IG than in the CG (Table 2). At 12 weeks, even after adjustment for humidity, temperature, and UVA in the region, the difference of the two groups in TEWL remained statistically significant (adjusted for humidity and temperature, *P* = 0.024; adjusted for UVA, *P* = 0.032; adjusted for temperature, high temperature, and ultraviolet A, *P* = 0.031) (Figure 2). In terms of skin hydration, more improvement was evident in the IG than in the CG, although there were no statistical significances in the differences of the change from the baseline between the two groups (Figure 2). After adjustment for humidity, temperature, and UVA in the region, TEWL was significantly different between the subjects over 50 years of age in both the CG and IG (Table 3).

**Table 2:** Comparison of changes in skin properties in the full analysis set population.

**Variable**	**Observed values**		**Changes from baseline**							
	**CTP (*n* = 36)**	**CG (*n* = 38)**	**CTP (*n* = 36)**	***P*^**a**^**	**CG (*n* = 38)**	***P*^**a**^**	***P*^**b**^**	***P*^**c**^**	***P*^**d**^**	***P*^**e**^**
**Transepidermal water loss (g/m**^**2**^**/h)**										
Baseline	6.08 ± 2.1	5.64 ± 1.4								
At 6 weeks	4.43 ± 1.6	4.25 ± 1.1	−1.65 ± 1.9	<0.001	−1.39 ± 1.9	<0.001	0.577	0.675	0.411	0.445
At 12 weeks	5.14 ± 1.5	5.31 ± 1.5	−0.94 ± 1.8	0.004	−0.33 ± 1.9	0.297	0.168	0.024	0.032	0.031
**Skin hydration (A.U.)**										
Baseline	192.5 ± 21.4	199.7 ± 21.9								
At 6 weeks	199.2 ± 19.6	200.1 ± 24.0	6.73 ± 22.0	0.076	0.45 ± 21.9	0.900	0.223	0.243	0.204	0.214
At 12 weeks	209.0 ± 18.7	212.1 ± 16.0	16.50 ± 21.7	<0.001	12.44 ± 21.7	<0.001	0.423	0.325	0.335	0.278
**Skin elasticity (mm)**										
Baseline	0.40 ± 0.11	0.42 ± 0.10								
At 6 weeks	0.35 ± 0.10	0.36 ± 0.10	−0.04 ± 0.1	0.050	−0.06 ± 0.1	0.002	0.574	0.514	0.557	0.642
At 12 weeks	0.33 ± 0.10	0.36 ± 0.09	−0.07 ± 0.1	<0.001	−0.05 ± 0.1	0.002	0.480	0.471	0.546	0.603
**Lt. crow's–feet visual score**										
Baseline	2.25 ± 0.73	2.47 ± 0.98								
At 6 weeks	1.94 ± 0.63	1.97 ± 0.88	−0.31 ± 0.6	0.003	−0.50 ± 0.6	<0.001	0.145	0.207	0.148	0.208
At 12 weeks	1.61 ± 0.60	1.71 ± 0.73	−0.64 ± 0.6	<0.001	−0.76± 0.5	<0.001	0.349	0.307	0.303	0.312
**Rt. crow's-feet visual score**										
Baseline	2.11 ± 0.78	2.24 ± 0.94								
At 6 weeks	1.61 ± 0.64	1.82 ± 0.83	−0.50 ± 0.6	<0.001	−0.42 ± 0.5	<0.001	0.524	0.417	0.512	0.431
At 12 weeks	1.44 ± 0.56	1.47 ± 0.65	−0.67 ± 0.6	<0.001	−0.76 ± 0.6	<0.001	0.514	0.497	0.537	0.564
**Lt. wrinkles by vision meter**										
Baseline	28.0 ± 6.2	25.8 ± 6.0								
At 6 weeks	31.4 ± 6.9	29.3 ± 5.4	3.33 ± 9.2	0.037	3.44 ± 8.3	0.011	0.960	0.797	0.985	0.897
At 12 weeks	29.8 ± 4.8	30.2 ± 6.1	1.82 ± 7.1	0.131	4.41 ± 7.9	0.002	0.141	0.181	0.241	0.286
**Rt. wrinkles by vision meter**										
Baseline	27.2 ± 6.0	27.9 ± 6.8								
At 6 weeks	29.3 ± 5.4	28.4 ± 6.1	2.15 ± 7.9	0.117	0.57 ± 8.0	0.663	0.406	0.390	0.334	0.395
At 12 weeks	29.3 ± 5.0	30.5 ± 7.0	2.08 ± 7.4	0.100	2.66 ± 8.6	0.065	0.758	0.894	0.976	0.942

*Values are presented as mean ± SD*.

a *Paired t-test*.

b *Two-sample t-test*.

c *Repeated measures ANCOVA (adjusted for temperature and humidity)*.

d *ANCOVA (adjusted for ultraviolet A)*.

e *ANCOVA (adjusted for temperature, high temperature, and ultraviolet A)*.

**Table 3:** Comparison of changes in skin properties in over 50 years of age.

**Variable**	**Observed values**		**Changes from baseline**						
	**IG (*n* = 14)**	**CG (*n* = 19)**	**IG (*n* = 14)**	***P*^**a,b**^**	**CG (*n* = 19)**	***P*^**a,b**^**	***P*^**c,d**^**	***P*^**e**^**	***P*^**f**^**
**Transepidermal water loss (g/m**^**2**^**/h)**									
Baseline	6.60 ± 2.64	5.54 ± 1.61							
At 6 weeks	4.53 ± 1.25	3.97 ± 0.97	−2.07 ± 2.20	0.003^a^	−1.57 ± 2.19	0.002^a^	0.413^c^	0.152	0.294
At 12 weeks	5.39 ± 1.87	5.16 ± 1.12	−1.21 ± 2.00	0.056^a^	−0.39 ± 1.89	0.657^a^	0.209^c^	0.034	0.040
**Skin hydration (A.U.)**									
Baseline	192.39 ± 24.09	195.89 ± 25.70							
At 6 weeks	203.90 ± 21.82	192.92 ± 27.34	11.50 ± 19.57	0.047^b^	−2.97 ± 21.56	0.555^b^	0.057^d^	0.043	0.046
At 12 weeks	212.55 ± 19.33	212.42 ± 16.51	20.16 ± 21.55	0.004^b^	16.53 ± 22.70	0.005^b^	0.646^d^	0.622	0.639
**Skin elasticity (mm)**									
Baseline	0.40 ± 0.11	0.39 ± 0.10							
At 6 weeks	0.38 ± 0.12	0.37 ± 0.09	−0.03 ± 0.13	0.485^b^	−0.03 ± 0.07	0.124^b^	0.986^d^	0.931	0.720
At 12 weeks	0.35 ± 0.11	0.37 ± 0.10	−0.05 ± 0.10	0.079^b^	−0.02 ± 0.07	0.208^b^	0.316^d^	0.316	0.330
**Lt. crow's**–**feet visual score**									
Baseline	2.57 ± 0.65	3.00 ± 0.88							
At 6 weeks	2.29 ± 0.47	2.47 ± 0.90	−0.29 ± 0.61	0.219^a^	−0.53 ± 0.61	0.004^a^	0.353^c^	0.287	0.447
At 12 weeks	1.93 ± 0.62	2.00 ± 0.82	−0.64 ± 0.63	0.012^a^	−1.00 ± 0.47	<0.001^a^	0.094^c^	0.033	0.039
**Rt. crow's-feet visual score**									
Baseline	2.43 ± 0.76	2.68 ± 0.95							
At 6 weeks	1.93 ± 0.73	2.26 ± 0.87	−0.50 ± 0.52	0.016^a^	−0.42 ± 0.51	0.008^a^	0.673^c^	0.731	0.506
At 12 weeks	1.71 ± 0.61	1.79 ± 0.71	−0.71 ± 0.61	0.004^a^	−0.89 ± 0.66	0.0001^a^	0.446^c^	0.414	0.471
**Lt. wrinkles by vision meter**									
Baseline	28.16 ± 7.87	26.58 ± 6.24							
At 6 weeks	30.71 ± 5.56	28.59 ± 5.34	2.56 ± 10.12	0.330^b^	2.01 ± 8.18	0.298^b^	0.865^d^	0.607	0.740
At 12 weeks	30.23 ± 4.06	30.04 ± 5.65	2.07 ± 7.42	0.315^b^	3.46 ± 8.74	0.081^b^	0.634^d^	0.891	0.973
**Rt. wrinkles by vision meter**									
Baseline	28.33 ± 6.09	28.12 ± 6.92							
At 6 weeks	29.03 ± 6.12	27.63 ± 6.60	0.70 ± 9.24	0.764^b^	−0.48 ± 8.71	0.811^b^	0.709^d^	0.536	0.548
At 12 weeks	30.16 ± 6.38	30.72 ± 7.47	1.83 ± 9.22	0.445^b^	2.60 ± 9.21	0.418^a^	0.814^d^	0.955	0.870

*Values are presented as mean ± SD*.

a *Wilcoxon's signed rank test*.

b *Paired t-test*.

c *Mann–Whitney's U test*.

d *Two-sample t-test*.

e *Repeated measures ANCOVA (adjusted for ultraviolet A)*.

f *ANCOVA (adjusted for temperature, high temperature, ultraviolet A)*.

#### Self-Assessment by Participants

Subjects in both groups reported that their skin felt a bit better over time, with no significant difference between the groups (Table 4). They reported no unpleasant skin sensations after the 12-week oral consumption of the supplements (Table 4). But, in the subgroup analysis, subjects under 50 years of age showed a significant improvement in total score and moisture in the subjective skin improvement questionnaire after taking CTP for 12 weeks (*P* < 0.05, data not shown), and subjects over 50 years of age reported that the tightness was significantly improved compared to the control group after taking CTP for 6 weeks (*P* < 0.05, data not shown).

**Table 4:** Self-assessment of skin texture in per protocol analysis.

**Variable**	**Intervention group (*****n*** **=** **36)**				**Control group (*****n*** **=** **38)**					
	**6 week**	**12 week**	**Δ**	**P^**a**^**	**6 week**	**12 week**	**Δ**	**P^**a**^**	**P^**b**^**	**P^**c**^**
Total score	35.22 ± 3.4	35.75 ± 3.6	0.53 ± 2.7	0.256	34.87 ± 4.1	35.45 ± 4.4	0.58 ± 4.8	0.461	0.748	0.719
Hydration	3.42 ± 0.6	3.50 ± 0.6	0.08 ± 0.6	0.373	3.37 ± 0.6	3.50 ± 0.7	0.13 ± 0.8	0.303	1.000	0.969
Smoothness	3.36 ± 0.5	3.53 ± 0.6	0.17 ± 0.6	0.083	3.34 ± 0.7	3.53 ± 0.7	0.18 ± 0.8	0.147	0.992	0.911
Roughness	3.28 ± 0.6	3.47 ± 0.7	0.19 ± 0.6	0.070	3.42 ± 0.6	3.47 ± 0.7	0.05 ± 0.9	0.711	0.993	0.959
Glowing	3.36 ± 0.5	3.36 ± 0.5	0.00 ± 0.5	1.000	3.26 ± 0.7	3.32 ± 0.7	0.05 ± 0.7	0.644	0.766	0.843
Elasticity	3.39 ± 0.6	3.36 ± 0.5	−0.03 ± 0.5	0.744	3.34 ± 0.6	3.32 ± 0.7	−0.03 ± 0.8	0.845	0.749	0.756
Wrinkles	3.31 ± 0.5	3.25 ± 0.4	−0.06 ± 0.5	0.487	3.16 ± 0.5	3.29 ± 0.6	0.13 ± 0.6	0.169	0.739	0.914
Itching	2.97 ± 0.3	2.97 ± 0.6	0.00 ± 0.6	1.000	2.97 ± 0.4	2.97 ± 0.6	0.00 ± 0.8	1.000	0.992	0.923
Aching	3.00 ± 0.0	3.00 ± 0.0	0.00 ± 0.0	–	3.00 ± 0.4	2.95 ± 0.4	−0.05 ± 0.6	0.571	0.422	0.382
Burning	2.94 ± 0.3	3.00 ± 0.2	0.06 ± 0.2	0.160	3.00 ± 0.4	3.03 ± 0.4	0.03 ± 0.6	0.785	0.746	0.827
Stinging	2.97 ± 0.4	3.00 ± 0.4	0.03 ± 0.3	0.571	2.89 ± 0.3	2.92 ± 0.4	0.03 ± 0.5	0.734	0.383	0.366
Tightness	3.22 ± 0.6	3.31 ± 0.5	0.08 ± 0.4	0.263	3.11 ± 0.5	3.16 ± 0.6	0.05 ± 0.6	0.600	0.282	0.300

*Values are presented as mean ± SD*.

a *Paired t-test*.

b *Two-sample t-test comparing the change between 6 and 12 weeks*.

c *Repeated measures ANCOVA (adjusted for temperature, humidity and ultraviolet A)*.

### Safety

Most of the subjects completed the protocol without adverse symptoms. One subject in the CG complained of mouth dryness. This symptom was not determined to be unrelated with taking CTP. No clinical changes in the levels of liver enzyme, creatinine, and glucose were observed in either group. No intergroup differences were found in these parameters during the study period.

## Discussion

Although CTP has long been one of the most popular supplements for anti-skin aging, there is scarce evidence of its effect on humans. This clinical trial aimed to confirm the efficacy, safety, and tolerability of CTP on various skin parameters. Our results showed that daily oral intake of 1,000 mg CTP for 12 weeks decreased TEWL and was tolerated among middle-aged women. Skin condition is determined by a combination of factors that include age, sex, and measurement site, and also lifestyle and skin physiology (27). Skin moisturizing properties and UV exposure are higher in spring and summer seasons than in the dry autumn and winter seasons (28). UVA can penetrate deep into the skin and damage blood vessels and intradermal cell infiltration (29–31). Therefore, we adjusted for external weather factors to specifically assess the efficacy of CTP intake among subjects. After adjustment for humidity, temperature and UVA of the region in which the participants lived during the trial, the difference in TEWL between the two groups remained significant.

Aging is associated with a reduction in skin thickness and in the number of epithelial cells, with a concurrent decrease in stromal collagen (2, 3). Compared to younger women, the skin aging process in middle-aged women may intensify as estrogen deficiency is reported to have a direct effect on the epidermis (32). Also, many middle-aged women are reported to be very concerned about their facial appearance and visible aging phenomena indicated by increasing expenditures for cosmetic products and aesthetic procedures (33). In this context, our findings are clinically important as the study included middle-aged women who could be expected to benefit most from collagen treatment compared to men or younger women.

The biological underlying mechanism of efficacy of collagen in skin function can be explained by its action in the dermis (34). However, it is clinically challenging to make collagen bioavailable so that it reaches the target tissue and participates in various physiological functions, since collagen is a large molecule and is rarely cleaved in the gastrointestinal tract into a bioactive form (35). This is why there is relatively insufficient evidence of the effect of collagen on skin, even though it is one of the most commonly used ingredients of skin health supplements (4, 21, 36). In an effort to enhance the biological activity of collagen on skin, LMWCP has increasingly been manufactured. The smaller peptide is expected to better tolerate the action of gastrointestinal and plasma enzymes, and will pass across the membranes of intestinal epithelial cells (15).

The CTP used contained 3.2% Gly-Pro-Hyp. This sequence is a major active constituent of collagen-derived peptides (37). Pharmacological bioavailability research has revealed that the Pro-Hyp dipeptide that is derived from Gly-Pro-Hyp is available at high concentrations in the human blood stream for several hours after oral administration (35, 38). In an animal study, it was demonstrated that 14C-labeled Pro-Hyp reaches the skin and bone tissues rapidly after ingestion (39). A more recent animal study found that orally administered collagen peptide protects against UVB-induced skin aging through the absorption of Pro-Hyp (40). Moreover, a clinical study identified Pro-Hyp in urine after collagen hydrolysate intake, suggesting that these two types of collagen are relatively stable and resistant to peptidases in the blood stream, and can reach the skin tissues (13, 41). Additionally, some *in vitro* studies that investigated the physiological function of Pro-Hyp in skin dermal fibroblasts reported that Pro-Hyp enhanced cell proliferation activity and stimulating the chemotaxis of dermal fibroblasts (34). Additionally, Pro-Hyp facilitated the production of hyaluronic acid in dermal fibroblasts (42). These physiological roles of Pro-Hyp are important to improve the efficacy of collagen hydrolysates in maintaining skin health. Presently, the beneficial outcomes of the oral intake of collagen on the assessed skin conditions suggests that Gly-Pro-Hyp and Pro-Hyp contained in CTP may be efficiently absorbed and biologically active.

The majority of clinical studies of CTP have reported positive results concerning the overall improvement in skin texture after supplementation with CTP (4, 15, 36, 43–45). However, the collagen supplements used in these studies included other ingredients that boost collagen production or prevent its degradation, such as antioxidants containing vitamins or minerals (46). Therefore, the increase in skin elasticity might be attributed not only to the presence of the collagen bioactive peptides, but also to the presence of a mix of antioxidants. Since the beneficial properties of these ingredients are well-known and have been extensively studied (47), previous results are inconclusive concerning whether CTP itself has a favorable effect on skin properties and the degree of improvement. The present study used a supplement containing only CTP in the test group to specifically investigate the effect of CTP on skin.

With respect to the property of CTP used, a recent randomized control trial was comparable to our study (15). The trial compared the study group given 1,000 mg of LMWCP (containing more than >15% tripeptide content including 3% Gly-Pro-Hyp) and 100 mg of vitamin C with the placebo group that received 100 mg of vitamin C only. This study demonstrated that the 12-week oral intake of LMWCP improved hydration, elasticity, and wrinkling among females 40–60 years of age. However, the positive outcomes may have resulted from the different weather conditions in which parameters of skin properties of the participants were tested, considering that the study was conducted from February (winter with low humidity and cold temperatures in the country in which the study was carried out) through June (summer with high humidity and temperatures). Even though the authors tried to exclude the effect of atmosphere on skin texture by having the participants remain in a constant temperature (22–24°C) and humidity (40–60%) room for 30 min prior to the assessment, the skin conditions of the participants must have been affected by the overall weather conditions at the two different time points of the evaluation. Environmental humidity, temperature, and UVA affect skin barrier functions (31). Climatic factors, such as cold temperatures and low humidity, accelerate water loss from the skin and increase the risk of skin dryness and dermatitis (31).

In that sense, clinical studies related to changes in skin texture should include the seasonal variations of the region where the intervention were performed as a major covariate factor. In an effort to exclude the effect of the weather conditions on skin properties, we adjusted for humidity, temperature, and UVA of the region in which our study participants lived during the study period in the analysis of the changes in skin parameters after 12 weeks of oral intake of CTP. As the average humidity, temperature and UVA were obtained from the local weather office, the environmental data were reliable and accurate. This adjustment for the weather conditions might partially explain the reduced improvements in skin parameters in our study participants compared to the participants of the previous study (15). Additionally, we assumed that a significant improvement in skin conditions in our placebo group might be resulted from seasonal changes. Before our study, there have been a number of studies showing the positive outcomes of CTP application in skin health (4, 15, 36, 43–45). However, no one had considered climatic factors, which distinguishes our study from previous research.

Owing to the low temperature and/or high salt in the surrounding environment, fish are a good source of collagen compared with collagen peptide derived from land animals. The fish-derived collagen features unique molecular and biological properties of amino acid composition and antioxidant, anti-skin aging and anti-fat accumulating activities (10, 36, 48). Ohara et al. (49) compared quantity and structures of food-derived gelatin hydrolysate peptides in human blood from different sources (gelatin hydrolysis of fish and porcine) of type 1 collagen. In the case of free form Hyp, fish gelatin hydrolysate was more absorbed than porcine gelatin hydrolysis suggesting that low molecular collagen from fish gelatin can be greater at improving bioavailability and skin health. Moreover, in comparison with mammalian-based collagen, the utilization of marine-based collagen is growing fast due to its unique properties such as no risk of transmitting diseases, a lack of religious constraints, a cost-effective process, low molecular weight, biocompatibility, and its easy absorption by the human body.

To increase the bioavailability of bioactive peptides, the CTP used presently was prepared from fish skin of Tilapia by digestion method using collagenase from *Bacillus*, non-pathogenic bacteria of the genus *Bacillus*. The Gly-Pro-Hyp and Pro-Hyp constituents of the CTP were better absorbed and attained higher plasma levels after oral administration in rats compared to high molecular weight collagen peptide in an animal study; Gly-Pro-Hyp and Pro-Hyp were stable in gastrointestinal fluid and rat plasma for 2 h, and Gly-Pro-Hyp was able to be transported across the intestinal cell monolayer (14). Furthermore, a more recent histological study analyzed the expressions of biomarkers in mice to evaluate the cutaneous hydration effect of oral intake of this CTP product (28). Increased expression levels of ceramide kinase, hyaluronic acid, collagen 1A, and hyaluronan synthase-2 (HAS2), and decreased levels of hyaluronidase-1 (HYAL1) and CD44 were observed in human derma fibroblasts after application of CTP. Furthermore, significant reductions of TEWL, scratching behavior, HYAL1, tumor necrosis factor-alpha (TNF-α), and interleukin-6 (IL-6), and increased water content and HAS2 levels were observed. These results suggested that CTP product used in our study could enhance skin hydration and have potential as a skin hydration agent in humans. Fish is becoming a popular ingredient in both raw and cooked form because of its nutritional value and reduced caloric intake. As a result, a large amount of fish skin is currently disposed. From an environmental perspective, making better use of by-products such as skin is prudent.

The present study has several limitations. We conducted at a single center and included only females, which prevents generalization of the results. Due to small sample size and short study duration, the difference of the effect of collagen on skin properties between the two groups was modest although it was statistically significant. Also, it could be difficult to extend all of our considerations to climatic conditions. Lastly, we used collagen pills in order to offer the same flavor, color, and taste to the two groups as a double-blind RCT. However, it is known that liquid collagen is absorbed into the bloodstream more quickly and efficiently and shortens the digestion time of protein than do solid supplements. Despite these limitations, our study is considerably valuable owing to the following strengths. Firstly, to the best of the authors' knowledge, it is the first clinical study to examine the efficacy of CTP on skin properties after adjustment for the weather conditions of the area where the subjects lived during the period of the study using accurate data from the local meteorological office. This study was a rigorously conducted randomized controlled trial, with the appropriate criteria for inclusion/exclusion. Moreover, the measurements of skin parameters were checked objectively by using a reliable quantitative method.

In conclusion, this randomized, placebo-controlled clinical trial demonstrates that CTP ingestion is well-tolerated and helps reduce water loss in in middle-aged women. Our results also provide initial insight into the effects of the climate data on the temperature, humidity, and UVA of the region in which the participants lived on skin properties in humans, and suggest that, in particular, the weather effects of the skin intervention on human skin health should be considered in future clinical trials with a population, especially in areas with four seasons.

## Data Availability Statement

The raw data supporting the conclusions of this article will be made available by the authors, without undue reservation.

## Ethics Statement

The studies involving human participants were reviewed and approved by The Ethics Committee of the Institutional Review Board at Pusan National University Yangsan Hospital. The patients/participants provided their written informed consent to participate in this study.

## Author Contributions

YJT, YLL, H-CK, and SYL contributed to the conceptualization of the study. DKS, AHK, JIK, YLL, H-CK, and SYL designed the methodology of the work. YLL and SYL had an active role in the process of participants and data acquisition. YJT and SYL contributed to the validation of results. DKS, AHK, and JIK carried out the formal analysis of the data. YJT and AHK worked together for data curation. YJT, Y-WK, and SYL wrote the work's draft and reviewed the final document. SYL coordinated and supervised the entire project. All authors contributed to the article and approved the submitted version.

## Conflict of Interest

DKS, AHK, and JIK is currently employed by Amicogen Inc. Biotech R & D center. The remaining authors declare that the research was conducted in the absence of any commercial or financial relationships that could be construed as a potential conflict of interest.
